# Pregnant at the start of the pandemic: a content analysis of COVID-19-related posts on online pregnancy discussion boards

**DOI:** 10.1186/s12884-022-04802-z

**Published:** 2022-06-16

**Authors:** Rebekah Choi, Ashwini Nagappan, Deena Kopyto, Anna Wexler

**Affiliations:** Department of Medical Ethics and Health Policy, University of Pennsylvania, Blockley Hall, 423 Guardian Drive, Philadelphia, PA 19104 USA

**Keywords:** Pregnancy, COVID-19, Anxiety, SARS-CoV-2, Online discussion forums, Online forums, Public health, Qualitative research, Pandemic, Digital health

## Abstract

**Background:**

A growing body of evidence indicates that the COVID-19 pandemic has had detrimental mental health effects for pregnant women. However, little is known about the specific stressors that increased anxiety for pregnant women at the start of the pandemic. The present study aimed to better understand the concerns of pregnant women during the beginning COVID-19 pandemic by analyzing content posted during the month of March 2020 on online pregnancy message boards hosted on WhatToExpect.com.

**Methods:**

All posts published between March 1–31, 2020 on nine different due-date specific WhatToExpect.com message boards were reviewed for COVID-19 relevance. Posts mentioning COVID-19 or its direct effects (e.g., “quarantine” or “stay-at-home order”) were included in our final sample. Data were coded by three authors according to a codebook developed inductively by all four authors. Posts were analyzed by overall frequency of appearance, by trimester, and temporally across the month of March 2020.

**Results:**

Across the 5,541 posts included in our final sample, the most common topics were fear of COVID-19 exposure, concerns with labor and delivery, navigating social interactions, and disruptions to prenatal care. The most dominant topics by trimester were disruptions to prenatal care (first trimester), fear of COVID-19 exposure (second trimester), and concerns about labor and delivery (third trimester).

**Conclusion:**

Our findings add to prior literature by demonstrating the salience of social concerns, which was the third largest COVID-19 topic in our sample. Emotional distress was most salient with regard to restrictions on birthing partners, but was apparent in everything from disruptions to pregnancy announcements, to cancelled baby showers, and limitations on newborn visitors. Given that anxiety during pregnancy is associated with worse maternal–fetal health outcomes, in the early stages of future pandemics healthcare providers should focus not only on strictly health-related concerns expressed by pregnant women, but also more broadly on other sources of anxiety that may be impacting the well-being and mental health of their patients.

**Supplementary information:**

The online version contains supplementary material available at 10.1186/s12884-022-04802-z.

## Introduction

A significant body of literature has demonstrated the detrimental mental health effects the COVID-19 pandemic has had on pregnant women. In studies conducted across different international contexts, largely in the early stages of the pandemic, pregnant women consistently reported a high prevalence of anxiety and depressive symptoms [1–17]. One review of the literature found rates as high as 58% for depression (in Spain) and 72% for anxiety symptoms (in Canada) amongst pregnant women [18]. The most comprehensive literature review published to-date on mental health and pregnancy during COVID-19, which encompassed 81 publications, concluded that there were “increased psychological symptoms, especially depressive and anxiety symptoms, in pregnant and postpartum women during COVID-19” [19].

Given that anxiety during pregnancy is associated with adverse maternal–fetal health outcomes [20–24], several studies attempted to obtain a deeper understanding of the driving factors of anxiety during COVID-19. This research found that concerns about childbirth, family members getting COVID-19, having a good birthing experience, and disruption of routine care all represented significant sources of stress [3, 25–28]. However, these studies have had limitations: many have been structured surveys, often administered via the Internet, with categories of concern prepopulated by researchers; most also utilized retrospective recall of mental health symptoms, which has a high risk of bias [19]. Thus, the overall context of anxiety during pregnancy has not been clearly or comprehensively elucidated.

An alternative method of assessing COVID-19-related concerns is to examine online content that is generated via social media and discussion boards. This approach is advantageous in that it utilizes text that has been generated in a naturalistic context, and provides a degree of temporal specificity (i.e., posts can be mapped to precise timepoints) that is absent from more traditional quantitative and qualitative methods. To date, many studies have characterized COVID-19 concerns on Twitter, Reddit, and other online fora [29–39], but only one—a content analysis of a Japanese question-and-answer website—specifically examined concerns of pregnant women during the COVID-19 pandemic [40].

Online discussion boards related to pregnancy, such as those hosted by websites like WhatToExpect.com and BabyCenter.com, have become popular outlets for expectant mothers to seek healthcare information and emotional support from a virtual community of their peers [41, 42]. Because women often join specific discussion boards based on their due date, prior studies examining these fora—using both automated topic-modeling and manual qualitative analysis—have mapped the specific issues that women most commonly discuss in each trimester of pregnancy [43, 44].

Building on this literature, the present study aimed to better understand the concerns of pregnant women at the beginning of the COVID-19 pandemic by analyzing content on online discussion boards hosted on WhatToExpect.com. We sought to identify general areas of COVID-19 concern, as well as frequently appearing COVID-19 topics within each trimester, to gain a deeper knowledge of how pandemic-related worries may have varied for women at different stages of pregnancy at the start of the COVID-19 pandemic.

We chose to focus on posts made on WhatToExpect.com during the month of March 2020 for several reasons. First, this website caters to women from English-speaking countries (primarily the United States and Canada), where the most dramatic initial social and travel restrictions (i.e., travel bans and lockdown regulations) occurred in March 2020 [45, 46]; thus we expected to observe the greatest changes in the content of posts throughout this month. Second, since many prior studies of pregnant women’s mental health gathered data from early in the pandemic, and specifically during March 2020 [3, 8, 15, 47–49], we chose this timeframe so that our data would both complement and yield greater insight into this prior literature. Third, as March 2020 was the timepoint of greatest uncertainty for pregnant women in English-speaking countries as the COVID-19 pandemic initially unfolded, we expected that data from our study would yield information that might be generalizable to early stages of future pandemics.

## Methods

Our dataset consisted of all COVID-19-related posts published on nine different WhatToExpect.com online discussion boards between March 1 and March 31, 2020. We chose the website WhatToExpect.com (owned J2 Global, Inc.) because it hosts one of the most active discussion boards for English-speaking women [43] and all posts on the platform are publicly viewable. While the website does not display demographic information about its users, in previous research, the majority appeared to be from the United States, with a smaller number from other English-speaking countries such as Canada, the United Kingdom (UK), and Australia [43].

The most popular fora on the site are due date specific message boards (e.g., “November 2020 Babies Group”) where women can engage with a virtual community experiencing a similar chronological pregnancy journey. Because we sought to capture data from women in each of the three trimesters, we chose nine different due date message boards that roughly corresponded to the first trimester (September, October, November 2020 discussion boards), second trimester (June, July, August 2020 discussion boards), and third trimester (March, April, May 2020 discussion boards) during the March 1 – 31, 2020 time period of interest [43].

All posts made during the month of March 2020 on these nine message boards were reviewed by three authors (AN, DK, RC) for COVID-19 relevance. Posts that mentioned COVID-19 in the title or text of the post (e.g., “pandemic,” “coronavirus,” “COVID-19,” “covid,” and “corona”) or had any mention of direct COVID-19-related effects (“quarantine,” “self-quarantine,” “stay-at-home order,” “social distancing,” etc.) were included in our study. In cases where the posts did not explicitly mention COVID-19 but alluded to it (e.g., “What question should I be asking my doctor with everything going on?”), the post was reviewed by all three authors and included if it was unanimously judged to be related to COVID-19.

Qualitative data analysis was conducted according to the principles of grounded theory [50], with codes (i.e., topics) developed inductively by three authors following an examination of a subset of posts. Codes were iteratively revised in a process that involved reviewing additional data and revising code definitions [51]. Next, all four authors employed the process of code-mapping [52], whereby codes are combined and recategorized, to create both main codes (topics) and various subcodes (subtopics). For example, a post expressing concern about being induced early due to COVID-19 was coded to the subtopic “changes to planned birth schedule” within the overall umbrella topic of “labor and delivery.”

Each author reviewed approximately one-third of the dataset and assigned each forum post to a single code. To ensure that all three authors were employing codes consistently, a random subset of 200 posts was separately coded and reviewed by all three authors. Results were compared and discrepancies were resolved through discussion among the authors. Three-way agreement was 74% (two-way interrater reliability across posts averaged 92.1%). All codes were entered and cleaned in Excel (Microsoft Corp., Redmond, WA, USA). Codes were analyzed by frequency of appearance, both overall (i.e., how often a given topic appeared across the entire dataset) and within trimesters (i.e., how often a give topic arose amongst posts from women in their first, second, or third trimester). Codes were also analyzed for variation over time through the month of March 2020; this temporal analysis was conducted using Tableau (Tableau Software, Seattle, WA, USA). This study was deemed exempt from review by the University of Pennsylvania Institutional Review Board as it did not meet the regulatory definition for human subject research and used publicly available data.

## Results

Approximately 60,600 posts were published between March 1 and March 31, 2020 across the nine groups included in our sample. Of those, 5,541 met our COVID-19 inclusion criteria and comprised our final sample. In line with our previous work examining these fora, it was our impression that the vast majority of posters were based in the United States, with a smaller number from other English-speaking countries. Nearly all posts appear to have been made by women, and there were frequent mentions of husbands and boyfriends but an absence of references to same-sex partners.

**Fig. 1:**
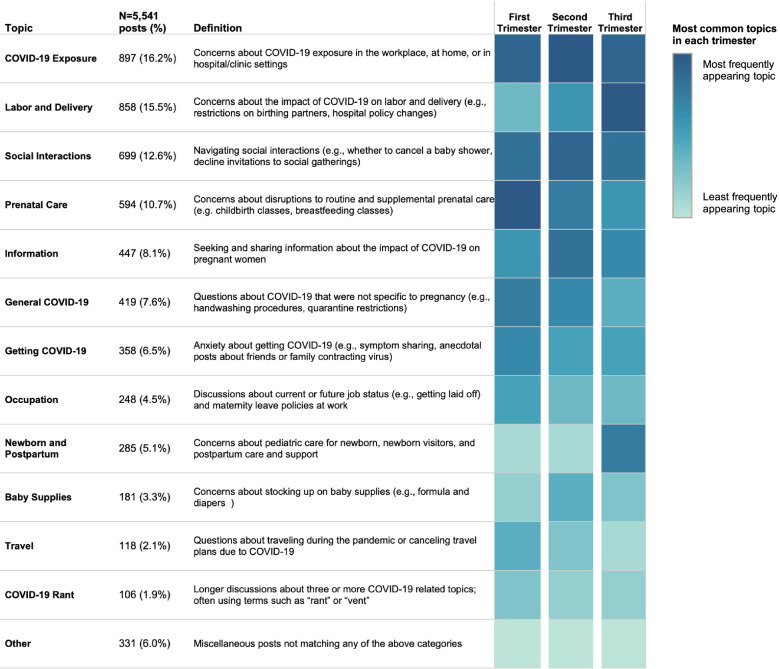
Most common COVID-19-related topics discussed on WhatToExpect.com online message boards. Main topics across all 5,541 posts are shown at left, sorted by overall frequency of appearance. The most frequently appearing topics within each of the three trimesters are shown in the heat map, with dark blue representing the most common topic within each trimester and light blue representing the least common

Figure 1 presents a detailed view of the most common COVID-19-related topics sorted by overall frequency of appearance. Overall, the largest number of posts across all trimesters were related to concerns about exposure to COVID-19 (16.2%). As shown in Table 1, which depicts subtopics and sample posts, the most common among these were fears about being exposed at work, with many posts coming from healthcare professionals or educators who did not have the option to work remotely. Amongst women in the first trimester, many posts were related to revealing the pregnancy to a supervisor earlier than they had planned. Other concerns in this topic centered on fear of being exposed to COVID-19 in medical settings like hospitals and doctors’ offices (e.g., wondering whether to “risk going to the clinic” for prenatal care) or being exposed at home due to family members’ behaviors (e.g., their husbands were “not taking COVID-19 seriously” by seeing friends at bars or going golfing).

**Table 1:** Subtopics and sample posts for COVID-19 exposure topic

**Topic**	**Subtopic** (n and % within topic)	**Sample Post**
**COVID-19 Exposure** (*N* = 897 posts)	*Occupational exposure* (*n* = 463; 51.6%)	I’m 13 weeks, and I’m a dental hygienist, which is one of the major risk jobs for transmission of coronavirus… Do you guys think I’m being too precautious if I don’t go into work?… I just wouldn’t forgive myself if something happened to my baby…
	*Family-related exposure* (*n* = 230; 25.6%)	I’m 13 weeks pregnant and struggling with the fact that my husband thinks the Coronavirus is just a “flu” and isn’t interested in social distancing whatsoever… aside from being exposed at work, he is continuing to go out with friends to breweries, etc.…. Am I totally out of line to be upset by the way he’s acting?
	*Hospital or clinic exposure* (*n* = 204; 22.7%)	I have an appointment tomorrow, I am 38 weeks and so scared of coming out of isolation and getting this exposure so close to baby time. Anyone not going or going to their doc appointments?

Posts about labor and delivery comprised the second largest topic (15.5%); most common were discussions related to disappointment about giving birth without the labor partners they had imagined would be present (Table 2). Women wrote about having to choose between their doula and their husband, having to exclude a close family member (e.g., their mother) from the delivery room, or having to give birth alone, either due to hospital policy or because of childcare constraints during COVID-19. These posts typically utilized highly emotive language, indicating shock (“I can’t believe I’m going to be forced to give birth by myself”), despair (“I just can’t stop crying”), and sadness (“I am really bummed my mom can no longer be part of the experience”).

**Table 2:** Subtopics and sample posts for labor and delivery topic

**Topic**	**Subtopic** (n and % within topic)	**Sample Post**
**Labor and Delivery** (*N* = 858 posts)	*Restrictions on birthing partners* (*n* = 238; 27.7%)	… I’m terrified if things get any worse before I go into labor… that they won’t allow my boyfriend (the father) to join me either. This situation is stressing me out beyond belief. I’m to the point where we are considering traveling to another hospital if mine places any more restrictions on visitors/support…
	*Hospital policy changes* (*n* = 225; 26.2%)	Hi everyone, I live in Brooklyn and my original plan was to deliver in one of the Brooklyn hospitals. But now due to Covid-19… I am scared and thinking to change my birthing hospital to nearby state. Is anyone of you thinking the same or have changed already and if so where are you going?
	*General concern about labor and delivery* (*n* = 143; 16.7%)	I am scared and terrified that when I go into labor… that this virus will be so much worse and I won’t be able to get the proper labor and delivery care or I’ll manage to get infected while at the hospital. I can’t see how other units won’t be affected if the hospital gets overwhelmed with covid 19 cases…This is so scary and I just feel angry that this is happening to so many moms to be like myself
	*Changes to planned birth schedule* (*n* = 93; 10.8%)	38w 3d today. I had my doctors appointment on Tuesday and asked to be induced at 39 weeks for many reasons, but one being I suffer from severe anxiety and with everything going on with coronavirus I’m terrified the hospital will change their rules on 1 support person. Anyways, got a call from my dr today saying they will not induce me. I’m pissed to say the least. Anyone else’s doctor NOT inducing them after 39 weeks? I’m so upset
	*Home birth due to COVID-19* (*n* = 71; 8.3%)	Hopefully this has settled down a bit by September but for now I’m definitely considering a planned home birth. Anyone else feeling this way?
	*Call to action* (*n* = 39; 4.6%)	http://chng.it/gVvxYbt2tL Please sign to safeguard mothers’ rights to have a support person with them in the hospital!
	*Arranging for childcare during labor* (*n* = 30; 3.5%)	…Given the current conditions surrounding COVID-19, I’ve come to accept that I will probably be delivering alone. We have a two year old that was originally going to stay with family; but now we’re more comfortable if my husband just watches her. My concern is actually after the delivery; I’ll be alone in the hospital with the baby… what if I’m exhausted and need to take a nap? Take a shower? Go to the bathroom?…
	*Specific concern about being separated from baby* (*n* = 19; 2.2%)	Hey ladies! So, I have heard and read that they may be separating mom from baby if they test positive for COVID-19 while in labor. I am currently 28 weeks pregnant and am terrified that this could possibly happen…

Discussions of hospital policy changes were another dominant subtopic in the labor and delivery category. Here, women sought general comparative information about labor and delivery policies in other hospitals, such as COVID-19 screening procedures, who was allowed in the delivery room, whether partners would be allowed to stay after delivery, and postpartum visitation protocols. Posts were both general (e.g., “What states or countries issued a rule of having no one with you when you deliver?”) and geographically specific (e.g., “Does anyone know the current rules for Australian hospitals?”). After two New York City hospital systems enacted a ban on labor partners on March 23 [53], women encouraged their peers to sign online petitions calling for the reversal of these policies. Other subtopics within the labor and delivery category included changes to a planned birth schedule (e.g., cancellations or postponements of scheduled inductions and Caesarian sections) and considerations of home births instead of hospital deliveries.

Concerns about how to navigate social interactions comprised the third largest topic (12.6%; Table 3), driven by a significant number of posts about baby showers made by women in the second and third trimesters. Towards the beginning of March, the majority of baby shower posts centered on women wondering whether they should cancel a planned shower. However, as the month progressed, the content of the baby shower posts shifted to queries about cancelling or postponing a shower (e.g., what wording to use in an email), the social etiquette surrounding gift-giving for a cancelled shower, and ideas for how to host a shower safely (e.g., online or socially distant). Other non-baby-shower related subtopics in this category included seeking information from peers on changes to social behaviors (e.g., wondering whether to keep kids from attending birthday parties and whether to allow friends into one’s home) and navigating the social aspects of disclosing a pregnancy earlier or differently than expected (e.g., announcing the news via video chat instead of in-person).

**Table 3:** Subtopics and sample posts for social interactions topic

**Topic**	**Subtopic** (n and % within topic)	**Sample Post**
**Social Interactions** (*N* = 699 posts)	*Baby shower* (*n* = 437; 62.5%)	Just spoke to my mom and MIL, and like so many of you we have decided to cancel the baby shower, and hopefully have a "sip & see" or other gathering later in the year after baby is born and health officials deem it safe to gather in large groups again. For those of you that have canceled, how did you do it—phone calls, email. letters in the mail?… Also curious to hear if you're doing anything virtual in place of the shower
	*Social behavior change* (*n* = 209; 29.9%)	One of my very best friends is getting married in 2 weeks but she’s having a very large wedding, 350 guests, & I’m considering not going because of the large crowd. Am I over reacting???
	*Considering disclosing pregnancy early due to COVID-19* (*n* = 35; 5.0%)	Hello! I’m 11 weeks pregnant and a dental assistant. With everything closed and being canceled due to this virus I’m feeling more and more guilty about going into work everyday! I obviously take the proper measures with wearing eye protection, gloves, a mask, and proper sanitizing techniques but still-does anyone think I should be worried enough to tell my boss I can’t come in anymore? I haven’t told him I am pregnant yet so I’m contemplating just telling him so he’s more understanding of why I can’t work during all this nonsense
	*Navigating social interactions – other* (*n* = 18; 2.6%)	This young couple gets into my elevator and stands like two feet away from me. I know I’m just Preggo and sensitive and pissy, but it’s really hard when you have kids and you’re homeschooling because your state is shut down and you are taking every precaution to be safe for your family and hospital staff and people without concerns are just like “oh whatever, doesn’t affect ME.” Like I’m literally holding my breath in an elevator after having my last two prenatal appointments cancelled because it is too risky to even BE THERE at ALL because people are too ignorant or selfish to think about how they’re affecting other people. Thanks for coming to my Ted Talk

Prenatal care comprised another main topic of concern (10.7%); most posts centered on the cancellation or postponement of routine in-person appointments and ultrasound scans (Table 4). Some women wondered about the utility of virtual appointments (e.g. “What can they even check if the whole appointment is online?”) and missed the reassurance that in-person appointments provided (e.g., “Doctor called and told me not to come into my appointment next week. How will I know my baby is okay?”). Many women noted that they had not seen their doctors in a while. In addition to disruptions to routine prenatal care, women discussed interruptions to supplemental prenatal care (e.g., cancelled childbirth classes, breastfeeding classes, and hospital tours), and a subset expressed concern about the worsening of a preexisting mental health condition due to the combined effect of pregnancy and COVID-19.

**Table 4:** Subtopics and sample posts for prenatal care topic

**Topic**	**Subtopic** (n and % within topic)	**Sample Post**
**Prenatal Care** (*N* = 594 posts)	*Concerns or issues with routine prenatal care* (*n* = 402; 67.7%)	Early anatomy scan cancelled due to coronavirus. The office said that they are now limiting the amount of scans to reduce traffic to the office and they wouldn't be giving the early anatomy scan. This is all due to the coronavirus… I'm super bummed. This is my rainbow baby and I'm a nervous wreck. The idea of going 9 weeks without a scan is nerve wracking. I'm not sure what to do. Should I beg the doctor to see if I they will give me the early scan?
	*Concerns or issues with supplemental prenatal care* (*n* = 140; 23.6%)	So our antenatal classes have understandably been cancelled and we're supposedly getting slides covering all the content that would've been included, which is great. However, I had planned on attending a breastfeeding workshop too. I'm already feeling pretty negative about breastfeeding because of all the horror stories I've heard, but I'm determined to give it a go, but I feel without this workshop I'll be at a disadvantage and more likely to give up. Can anyone advise anything in the UK that's helped them prepare, and that's also free? I've had to stop work as a self-employed person, so we're a salary down and can't really afford to pay for any online classes
	*Mental health care during pregnancy* (*n* = 38; 6.4%)	I've always struggled with depression/anxiety since I was a teen but now at 31 weeks pregnant I feel like Im in an never ending hole of darkness and sadness
	*Concerns or issues with non-pregnancy related care* (*n* = 14; 2.4%)	Hey moms! I have a sinus infection and I’m not able to get ahold of my primary Dr. and my OB still hasn’t got back to me. My local urgent care is closed because of the dumb @$$ coronavirus. Does anyone know what I can take that’s over the counter and safe during pregnancy? I feel horrible 34w 4d

Within the topic coded as “Information” (8.1% of all posts; Table 5), the majority of posts were from women sharing information about the impact of COVID-19 on pregnancy, such as links to articles or guidelines (e.g., Center for Disease Control recommendations for pregnant women). Some noted that they were sharing links due to the lack of existing information about pregnancy and COVID-19. Other posts in this category were from women seeking health information (e.g., “Is hand sanitizer safe for pregnant women?” and “Does anyone know if pregnant women are at higher risk of contracting the virus?”). Posts coded to this topic were distinguished from those coded to the next most frequent topic, “General COVID-19” (7.6% of all posts) in that the former were pregnancy-specific, whereas the latter consisted of questions that could have been asked by anyone during COVID-19 (e.g., “What are you cleaning with and how often?” and “Am I allowed to go to the pharmacy during mandatory lockdown?”).

**Table 5:** Subtopics and sample posts for information topic

**Topic**	**Subtopic** (n and % within topic)	**Sample Post**
**Information** (*N* = 447 posts)	*Sharing information such as PSAs, links to guidelines, videos and practical information on COVID-19 and pregnancy* (*n* = 293; 65.6%)	I see a lot of pregnant moms asking questions about the safety of their babies in the midst of all this. I just wanted to share this article I saw since there is very little information to go on at this stage until things actually happen. https://www.theguardian.com/world/2020/mar/14/newborn-baby-tests-positive-for-coronavirus-in-london
	*Seeking information from peers on COVID-19 impacts on pregnancy* (*n* = 154; 34.5%)	Has anyone heard anything from their dr about how the coronavirus could affect pregnant women? Or has anyone been given advice from a dr to not go into work and be extra careful while pregnant?

Among the remaining topics, “Getting COVID-19” (6.5% of all posts; see subtopics and sample posts in Additional file 1) included discussion of friends or family members testing positive for COVID-19, sharing of potential symptoms of COVID-19, and anxiety about possibly being infected by the virus. “Occupation” (4.5%) consisted of women seeking advice for work-related planning in the context of COVID-19, such as maternity leave policies, getting laid off due to the pandemic, and managing finances. Posts coded to “Newborn and Postpartum” (5.1%) were dominated by questions of how to navigate the issue of newborn visitors; other subtopics included changes to newborn photoshoots, lack of postpartum support, and concerns with accessing pediatric care. “Baby Supplies” (3.3%) and “Travel” (2.1%) comprised other topic categories.

A subset of posts (1.9%) expressed multiple COVID-19 concerns; because these often were qualified with “I’m ranting now…” and “just need to vent”, they were coded separately under “COVID-19 Rants.” Miscellaneous posts (6.0%) were coded to the “Other” category; examples include discussing a “silver lining” to the pandemic, sharing funny stories and memes, and requesting that others post something positive.

The most frequently appearing topics within each of the three trimesters are shown in the heat map in Fig. 1 with dark blue representing the most common topic within each trimester and light blue representing the least common. Dominant topics varied by trimester: the impact of COVID-19 on labor and delivery was the most common topic amongst women in the third trimester, fear of COVID-19 exposure was the most frequent in the second trimester, and prenatal care was the most common in the first trimester. Across all trimesters, navigating social interactions and general exposure represented significant concerns.

**Fig. 2:**
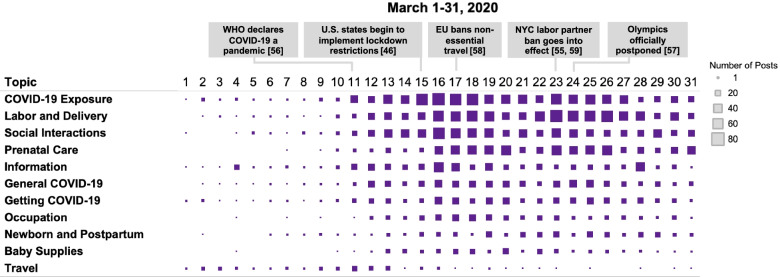
COVID-19 topic distribution mapped temporally across the month of March 2020. Note that topics with < 2% of posts and “Other” category are not depicted

Topic distribution was mapped temporally across all days in March 2020 (Fig. 2).

For all topics, there was little activity in the first ten days of March, aside from that related to media articles and travel-related questions. Posts gradually increased after March 11, peaking on March 16 with a daily total of 408 posts. The large increase in posts in the second week of March is correlated with the World Health Organization (WHO) officially declaring the outbreak a global pandemic on March 11 [54], as well as the rapidly increasingly recognition—driven by quarantine restrictions and cancellations of major sports and arts events—that a major public health crisis had emerged [45, 55, 56]. Some daily increases appear to have been tied to specific events: for example, the number of posts about delivering without a planned birthing partner peaked on March 23, the day that two New York City health systems banned labor partners in the delivery room [53, 57], and posts coded to “Information” peaked on March 16, when a *Daily Mail* article [58] about the UK’s proposed quarantine restrictions for pregnant women was widely shared across different message boards.

## Discussion

The present study provides a comprehensive overview of the COVID-19-related topics that appeared across 5,541 posts on online pregnancy-related discussion boards in March 2020, when the pandemic first became a national health emergency in English-speaking countries. In line with previous studies [2, 3, 5, 7, 8, 59], our results indicate that fear of COVID-19 exposure, impacts of the pandemic on labor and delivery, and disruptions to prenatal care were significant sources of concern at the start of the pandemic. Notably, our findings add to prior literature that assessed the mental health of pregnant women during early stages of the pandemic by demonstrating the salience of social concerns, as the third largest COVID-19 topic in our sample was related to navigating social interactions.

Our results also provide a glimpse into how, at the beginning stage of COVID-19, pandemic-related concerns varied for women at different stages of pregnancy. That impact to labor and delivery was the most common topic in the third trimester is in line with previous studies of online pregnancy forums, which has found that discussions of labor and delivery represent a disproportionately large share of posts [43, 44]. In the second trimester, where discussions of labor and delivery were less prevalent, the most dominant topic was fear of COVID-19 exposure. In the first trimester, impacts on prenatal care were particularly salient, with many women expressing concern about the health of their baby in the absence of in-person physician visits and ultrasound scans.

Across all topics, a recurring theme was that of disruptions to the imagined experience of pregnancy and childbirth—of women coming to terms with the reality of how the pandemic had suddenly and dramatically reshaped a momentous time period in their lives. The emotional distress was most salient with regard to restrictions on birthing partners, but disappointment and anxiety were apparent in everything from disruptions to pregnancy announcements, to cancelled baby showers, and limitations on newborn visitors. That pregnant women turned to online discussion boards to express frustration and seek support from their peers is not surprising, as prior literature has pointed to seeking emotional and social support as one of the primary reasons for participating in online pregnancy fora [43, 44, 60–62].

A secondary theme cutting across many of the topics in our study was how the pandemic removed established avenues of support and reassurance, such as in-person doctor’s visits, supplemental prenatal classes, and assistance from family and friends. Social support throughout pregnancy has been associated with better maternal health outcomes [63–65], whereas the lack of social support is a noted risk factor for poor pregnancy outcomes [66, 67]. The pandemic therefore created a doubly negative effect for women’s health: it both increased episodes that could lead to anxiety and stress, while at the same time removing the networks of social support that may have helped women cope more efficaciously [68].

This study has several limitations. First, we did not have access to demographic information such as age, gender, geographic location, and whether women were nulliparous vs. primiparous. As noted earlier, however, it was our impression that nearly all posts were made by pregnant women, the majority of whom appeared to be based in the United States. Second, all posts were coded to a single primary code for analytic purposes, even though in some cases posts may have touched upon more than one topic. Third, because we captured posts from March 2020, the COVID-19 topics that arose in our sample may not be reflective of COVID-19-related concerns at later timepoints, and COVID-19-related concerns of pregnant women may have shifted (or waned) since the start of the pandemic. Fourth, frequency of appearance of a topic does not necessarily reflect saliency; thus we have endeavored to note when specific topics used stronger emotional language. Finally, our findings may not be reflective of the concerns of all pregnant women, as it is unknown how those who post to online message boards may differ from those who do not.

By analyzing content created in a naturalistic context, rather than as responses to structured surveys, our findings add insight to the body of literature that has examined how the mental health of pregnant women is negatively affected during the early stages of pandemics [69]. Even though the COVID-19 pandemic is currently waning in many English-speaking countries and is likely approaching long-term endemicity [70–72], our results are relevant for future pandemics, as they demonstrate sources of anxiety and stress during early periods of uncertainty. It is crucial in future pandemics, therefore, that healthcare providers focus not only on strictly health-related concerns expressed by pregnant women, but also attend more broadly to other sources of anxiety that may be impacting the well-being and mental health of their patients.

## Supplementary information

**Additional file 1: Table S1**. Subtopics and sample posts for all additional topics listed in Figure 1 but not depicted in **Tables 1-5**, submitted as .pdf file.

## Data Availability

The datasets generated and/or analyzed during the current study are not publicly available due to the Terms of Use of WhatToExpect.com (https://www.whattoexpect.com/terms-of-use/), which prohibit reproduction or redistribution of content on their platform. However, the content of posts used in this study is publicly available, and examples of posts coded to each topic and subtopic are provided in Tables 1, 2, 3, 4, 5 and Additional file 1.
