# Supplementary material for: Pregnant at the start of the pandemic: a content analysis of COVID-19-related posts on online pregnancy discussion boards
Source: BMC Pregnancy Childbirth. 2022 Jun 16;22:493. doi: 10.1186/s12884-022-04802-z (PMC9201795; doi:10.1186/s12884-022-04802-z)
Supplement: Supplementary file 1 — Additional file 1: Table S1. Subtopics and sample posts for all additional topics listed in Figure 1 but not depicted in Tables 1-5, submitted as .pdf file. [file 12884_2022_4802_MOESM1_ESM.pdf]

Table S1. Subtopics and sample posts for all additional topics listed in Figure 1 but not depicted in Tables 1-5.

**Topic**	**Subtopic** (n and % within topic)	**Sample Post (edited for length)**
**General COVID-19** (N=419 posts)	*General COVID-19* (n=180; 43.0%)	How quarantined are you staying? Are you allowing visitors and how often?... We have been crazy about washing hands and I've been trying to keep clothes washed and things disinfected.
	Navigating lockdown and lockdown impacts (n=127; 30.3%)	So I live in MA, on the RI border and the panic over coronavirus around here is insane. I've heard rumors of shutting down the states completely and doing a mandatory quarantine like in Italy and Spain. My question and maybe it's dumb, but what does that mean for me going to the hospital to have my baby? I would have to cross state lines since my hospital is in RI (like 10-15 minutes from my house)
	Seeking quarantine activities (n=112; 26.7%)	Alright ladies i think we're all in the same boat with our minds being on overdrive from being close to delivery + most of us isolated from the pandemic. what are you ladies doing to distract your minds for a little?
**Getting COVID-19** (N=358 posts)	*General anxiety or concern about exposure to, or infection from, COVID-19* (n=225; 62.9%)	I'll be honest- this corona thing has me completely freaking out. i just cant have anything happen to my babygirl and so i'm completely paranoid, nearly have panic attacs if my husband goes to the supermarket...how do you stay calm?
	Do I or a loved one have coronavirus? (n=121; 33.8%)	Omg so I woke up today with body aches and chills so I took my temp and had a fever! Full on cried had a huge panic attack thinking I have corona and dr thinks its mastitis. Never thought I would be so thankful for mastitis! Hoping this corona thing is under control soon because my postpartum hormones and anxiety cant take it!!
	I have coronavirus (n=6; 1.7%)	I'm 18 weeks.. my doctor just told me i have covid 19 😩💔 I started with a cough last week.. it went from a cough to a cold to a runny nose..

		having trouble on breathing.. 😩 I lost my taste and smell on everything.. mommies please take care of yourself a this feeling sucks
	Family member or friend has COVID-19 (n=6; 1.7%)	Hey guys! I am in the United States and one of my good friends who lives in Spain has the coronavirus. Just wanted to come on here and post to reassure some of y'all who were expressing concern about it!... He hasn't even gone to the hospital and says the worst thing about it is the up and down fevers…The whole point of my post is just to hopefully help reassure some of you!! :)
**Occupation** (N=248 posts)	N/A	Me and my SO work for the same company, a mental health facility, who announced they were closing doors today for good due to COVID19… I feel very sad and very angry! We have LOST OUR JOBS!! Both of us!! On the very same day.
**Newborn and Postpartum** (N=285 posts)	*Newborn/infant visitors* (n=180; 63.2%)	Anyone letting grandparents come visit at your home? I'm so torn and it literally is making me cry about it. My parents did stop by yesterday to meet my new little one for the first time but now they want to come every other day. I would love them to do this but so scared because I don't want them to get sick. I was debating on just letting grandparents come by as long as they're healthy but hold off on everyone else. Thoughts?
	Newborn photoshoot (n=34; 11.9%)	I'm so sad. I know the world is crazy right now with the virus but I really wanted to have some pictures to capture this fleeting time in our lives. She's our third and last baby and I know how fast they change. I've tried to do some pictures on my own but they don't turn out very good and I wanted one with our whole family. Just feeling emotional and sad that I won't have pictures of this moment in our family's lives… Such a hard time to have our baby…
	Postpartum support	Hello! My husband and I are first time parents (two days past due) and we don't have family

	(n=32; 11.2%)	where we live. We had planned on having a PP Doula come to our home for the first couple weeks to assist with infant care, breastfeeding, etc. However, we are unsure what to do now…Is anyone still going to have a PP Doula that planned to have one? My husband and I have been working from home and social distancing for the last 2.5 weeks but we also realize that we may need this help for our sanity and learning how to best care for our child.
	Concerns with pediatric care for newborn (n=24; 8.4%)	Does anyone know if we still have to come in at babys check up when baby is days old, plus 2 weeks later? This whole covid-19 has me.not wanting to go to clinics at all…I really dont want to be going out alot during this time but I know it's better for us moms and baby to be checked upon. Anyone else concerned about this? and for you moms who already had your babies recently how many visits are you and will you have gone to since birth expected? Has the doctors said anything about waiting for a few weeks or so?
	Concerns with postpartum care (n=9; 3.2%)	I had my baby 2 weeks ago and now due to the covid virus I am not able to see my doctor for my 6 week check up…when is it okay to have sex and take baths again?
	Newborn care if COVID-19 positive (n=3; 1.1%)	I can't find any recommendations on what to do with newborn if we as mom or dad get sick. With limited testing. We may not know we have the corona virus... does anyone have any suggestions just to be prepared? Especially those who don't have family
	Mental health care after pregnancy/during postpartum period (n=3; 1.1%)	Any other mamas really struggling with PPD and PPA? I've been on Zoloft for three weeks now but honestly the only thing that really helps is getting out of the house and spending time with family….I'm really struggling. I know we can call and face time but it just isn't the same. 💔💔💔 I don't know how much longer my mental health can take quarantine

**Baby Supplies** (N=181 posts)	N/A	Is anyone else struggling to find diapers and wipes in stock anywhere? We've been using the Honest brand and I just realized you can order directly from their website. Lots of stuff in stock!! https://www.honest.com/baby-products/diapers-and-wipes
**Travel** (N=118 posts)	N/A	From March 13th: Much love to all you mommies out there in "danger zones" for covid-19. In a week I am supposed to travel to Tampa for vacation. I live in Ohio and I'm nervous about traveling on a plane or even driving the 20 hours just to spend 5 days on a beach. Just nervous in general. I'm 10 weeks along in my first pregnancy. Any advice or words of wisdom. I'm able to cancel the trip, just weighing my options.
**COVID-19 Rant** (N=106 posts)	N/A	…My family is constantly sending me articles on the daily about the virus mostly fake ones I should mention! about how pregnant women are most susceptible and my blood type (A) is at the "highest risk" and articles about newborns being born or dying from it. Like seriously why send me this *** and get me all worked up and worried?? … Now I'm even more heated because I woke up with a terrible sore throat NO other symptoms!!.... So now I have everyone on my ass telling me I need to go to the doctor ASAP and get checked for the virus 😑... But okay let me just stroll into my doctors office where I COULD potentially contract the virus just because I have one symptom 😑♀ …Sorry for the rant y'all I had to get it out because I'm literally boiling over today with all the stupidity.. Please tell me I'm not the only one fed up with humanity
**COVID-19 Accommodations** (N=18 posts)	N/A	So target and Kroger is allowing an extra hour in the morning for people over 60 and pregnant women! Target is only Tuesday's and Wednesday's and Kroger is Monday-Thursday!

**Other**<br>(N=313 posts)	N/A	Have so many things on my head with this whole Corona virus thing. I'm so over it want this to be over. Just want to take this time to send prayers to all. The ones that already had their baby and the ones still waiting may God protect all. God Bless you all.
